# Quantifying phase separation at the nanoscale by dual-color fluorescence cross-correlation spectroscopy (dcFCCS)

**DOI:** 10.52601/bpr.2022.210026

**Published:** 2022-02-28

**Authors:** Yirong Yao, Wenjuan Wang, Chunlai Chen

**Affiliations:** 1 School of Life Sciences; Beijing Advanced Innovation Center for Structural Biology; Beijing Frontier Research Center of Biological Structure, Tsinghua University, Beijing 100084, China; 2 School of Life Sciences; Technology Center for Protein Sciences, Tsinghua University, Beijing 100084, China

**Keywords:** Liquid-liquid phase separation, Nanoscale, Dual-color fluorescence cross-correlation spectroscopy, Molecular stoichiometry, Binding affinity

## Abstract

Liquid–liquid phase separation (LLPS) causes the formation of membraneless condensates, which play important roles in diverse cellular processes. Currently, optical microscopy is the most commonly used method to visualize micron-scale phase-separated condensates. Because the optical spatial resolution is restricted by the diffraction limit (~200 nm), dynamic formation processes from individual biomolecules to micron-scale condensates are still mostly unknown. Herein, we provide a detailed protocol applying dual-color fluorescence cross-correlation spectroscopy (dcFCCS) to detect and quantify condensates at the nanoscale, including their size, growth rate, molecular stoichiometry, and the binding affinity of client molecules within condensates. We expect that the quantitative dcFCCS method can be widely applied to investigate many other important phase separation systems.

## INTRODUCTION

Biomolecules are found to form membraneless organelles in live cells through multivalent interactions, which is known as LLPS (Banani* et al.* 2017; Hyman* et al.* 2014). A well-known membraneless organelle is nucleolus, which mediates RNA processing and ribosomal biogenesis (Boisvert* et al.* 2007; Feric* et al.* 2016; Mitrea* et al.* 2016). Many other condensates formed by LLPS also play important roles in various biochemical processes, including cell signaling and DNA damage responses (Wu 2013; Zeng* et al.* 2021). Recently, LLPS has attracted significant attention for its great importance in various cellular functions. Many studies are focused on the physical and chemical properties of condensates, mechanisms of condensates formation and the molecular basis of their functions (Alberti* et al.* 2019). Methods used in these studies include morphological, rheological and structural characterization of condensates (Mitrea* et al.* 2018). Optical microscopy, especially the confocal fluorescence microscopy, is the most commonly used method to visualize LLPS. However, it is restricted by the optical diffraction limit (~200 nm). Herein, we developed a dcFCCS method to capture condensates at the nanoscale. In addition, the dcFCCS can quantify the size, growth rate, molecular stoichiometry of condensates and the binding affinity with condensates.

## DEVELOPMENT OF NANOSCALE PHASE SEPARATION QUANTIFICATION METHODS

To detect phase separation at the nanoscale, various methods have been used in previous studies. Super-resolution microscopy has been applied to capture biomolecular condensates beyond the optical diffraction limit (Sydor* et al.* 2015). For instance, stochastic optical reconstruction microscopy (STORM), with a spatial resolution of ~30 nm, was used to observe stress granule *in vivo*, which revealed detailed characterization of core-shell structure of stress granules and quantified the size of cores (~200 nm in diameter) (Jain* et al.* 2016). In addition, transmission electron microscope was used to characterize the hydrogel formed by fused in sarcoma (FUS) protein, which is amyloid-like fibers with 500 nm in length and 30 nm in thickness (Kato* et al.* 2012). Correlated light and electron microscopy (CLEM) was also used to capture the ultrastructure of nucleolus (Normand* et al.* 2016). However, none of these techniques are able to capture the dynamic transition process from miscible individual molecules to micron-scale condensates. Here, we provided a detailed protocol of our previously published dcFCCS method (Peng* et al.* 2020). We believe that this protocol will help researchers to visualize and to quantify nanoscale condensates in other important LLPS systems.

## APPLICATIONS AND ADVANTAGES OF THE PROTOCOL

Our developed dcFCCS method is a simple and powerful way to quantify condensates at the nanoscale. Firstly, dcFCCS is suitable for freely-diffusing molecules, complexes and condensates, whose sizes range from sub-nanometer to micrometer. Therefore, the dynamic formation process from miscible individual molecules to condensates can be captured. Secondly, dcFCCS is highly sensitive to capture heterocomplexes and condensates containing two kinds of fluorophores. Therefore, concentrations far above the physiological conditions and the addition of crowding agents, which were commonly used to induce the formation of micron-scale condensates (Ferrolino* et al.* 2018), are not needed. Thirdly, molecular stoichiometry and binding affinity with condensates can be quantified. Lastly, for each data collection, only 5–20 μL of labeled molecules, whose concentrations range from sub-nmol/L to μmol/L, are needed, which is user-friendly. In our previous report, LLPS at the nanoscale formed from a model system built from yeast SmF (ySmF) variants was examined (Peng* et al.* 2020).

## LIMITATIONS OF THE PROTOCOL

Our method has the following limitations. Firstly, biomolecules of interest including proteins and nucleic acids need to be labeled with suitable organic fluorophores or be fused with fluorescent proteins, whereas commonly used turbidity experiment can use unlabeled samples. Secondly, dcFCCS calculates cross-correlation curves of two different fluorophores. When the formation of condensates is driven by three or more components, dcFCCS assays need to be performed and repeated with different doubly-labeling schemes. Three or four different fluorophores of different emission wavelengths can be used, only when their excitation and emission crosstalk is carefully corrected. Thirdly, dcFCCS can only capture freely-diffusing molecules and condensates. Therefore, large condensates, which precipitate, are not suitable for dcFCCS measurements. Lastly, to perform quantification analysis, size distributions of condensates cannot be too broad, so that the dcFCCS curve can be well fitted by the single-component diffusion model (Peng* et al.* 2020). In our ySmF LLPS model system, condensates formed at the nanoscale satisfy this criterion.

## OVERVIEW OF THE PROTOCOL

A common application of fluorescence correlation spectroscopy (FCS) is to quantify the dwell time of molecules of interest in the confocal detection volume, from which diffusion coefficients of molecules can be quantified (Hess* et al.* 2002). The rationale behind this protocol is based on the fact that the sizes of freely-diffusing molecules, complexes and condensates determine their diffusion coefficients, which can be quantified by the dcFCCS method (Schwille* et al.* 1997). Two fluorophores of different emission wavelengths are needed for dcFCCS assays, which not only measure the sizes of complexes and condensates containing both fluorophores but also quantify the proportions of labeled molecules participating in the condensates containing both fluorophores. Briefly, to perform dcFCCS measurements, components participating into LLPS formation are labeled. After preparation of passivated slides and calibration of dcFCCS instrument, dcFCCS measurements are performed to quantify LLPS at the nanoscale. After data analysis, the size and growth rate, the molecular stoichiometry of condensates can be quantified, as well as the binding affinity of client molecules within condensates. Note that some parts of the protocol can be modified and optimized, as described in the section “Experimental design” below.

## SUMMARIZED PROCEDURE

1　KKETPV_14_ and PDZ_14_, which are known to induce LLPS when mixing together, are incubated with maleimide derived Alexa Fluor 488 (AF488) and sulfo Cyanine5 (Cy5) at 1:5–1:10 molar ratio, respectively.

2　Reaction mixtures are incubated at room temperature for 2 h.

3　Labeled proteins are separated from excess free fluorophores through NAP-5 column.

4　Labeling efficiency is calculated by determining concentrations of proteins and fluorophores via *A*_280_, *A*_495_ and *A*_652_.

5　Aliquot labeled proteins and store at −80 °C after flash-frozen.

6　Prepare polyethylene glycol (PEG)-passivated slides.

(A) Slides and coverslips are sonicated at 40 °C through the treatment of ethanol, KOH and ethanol.

(B) Cleaned slides and coverslips are incubated with amino-silane solution at room temperature overnight.

(C) Silanized slides and coverslips are incubated with PEGylation solution at room temperature for 2.5–3 h.

(D) Slides and coverslips are washed, dried by clean N_2_, vacuum sealed and stored at −20 °C.

7　Standard dye sample is used to calibrate the excitation volume of dcFCCS.

(A) The confocal microscope used to carry out dcFCCS measurements is turned on, including software and laser.

(B) Place coverslips onto the microscope objective and adjust the focal point in the solution above the coverslips.

(C) Parameters of data acquisition are set.

(D) 10 nmol/L standard dye is applied to passivated coverslips.

(E) Raw photon data is recorded for 5 min under laser excitation.

(F) Autocorrelation curve of the standard dye is used to calculate the amplitude and diffusion time. Excitation volume is then calculated from diffusion time of the standard dye.

8　Dual-labeled DNAs are prepared to calibrate correction factors of dcFCCS instrument.

(A) Purchase AF488 labeled single-stranded DNA (ssDNA) and Cy5 labeled complementary ssDNA, which are then mixed at 1:4 and 4:1 molar ratio, respectively, and annealed to obtain double-stranded DNAs (dsDNAs) as dual-labeled standard samples.

(B) As Step 7(A), turn on the microscope.

(C) As Steps 7(B)–7(C), prepare coverslips, focus lasers into solution and set parameters for data acquisition.

(D) dsDNAs containing excess AF488 labeled ssDNA or excess Cy5 labeled ssDNA are diluted to 10 nmol/L and added to passivated coverslips, respectively.

(E) Raw photon data of AF488 and Cy5 detection channels is recorded for 5 min under excitation of both 488 nm and 640 nm lasers.

(F) Autocorrelation and cross-correlation curves are calculated to determine correction factors.

9　After sample preparation and dcFCCS instrument calibration, start to acquire the data of phase separation.

(A) Prepare the microscope, coverslips and control software as shown in 7(A)–7(C).

(B) dcFCCS curves and raw photon data of 10 nmol/L AF488-KKETPV_14_ or 10 nmol/L Cy5-PDZ_14_ under 488 nm or 640 nm laser excitation, respectively, are recorded for 5 min.

(C) AF488-KKETPV_14_ and Cy5-PDZ_14_ are mixed together to induce LLPS and the mixture is added to passivated coverslips. dcFCCS curves and raw photon data of the mixture are recorded for 9 min under 488 nm and 640 nm laser excitation.

(D) After additional 41 min incubation, dcFCCS curves and raw photon data of the mixtures are recorded for another 9 min under 488 nm and 640 nm laser excitation.

10 To create a phase diagram, different concentrations of KKETPV_14_ and PDZ_14_ are mixed. Repeat the Steps 9(C)–9(D).

11 PRM_14_ and Cy5 labeled (SH3-KKETPV)_14_ are mixed to form condensates in the tube.

12 Mix client protein, AF488 labeled PDZ, into the PRM_14_-(SH3-KKETPV)_14_ phase separation system, add the whole mixture to the coverslips and record dcFCCS curves and raw photon data for 5 min under 488 nm and 640 nm laser excitation.

13 dcFCCS curves and raw photon data are used to calculate the size, growth rate, stoichiometry of condensates formed, as well as the binding affinity of client protein within condensates.

(A) Quantify the size of condensates. The diffusion times ($\tau $_Dx_) derived from the cross-correlation curve are used to calculate hydrodynamic radii of condensates.

(B) Quantify the growth rate of condensates. Divide the 9-min-length raw data into 1-min-length data to recalculate dcFCCS curves, from which changes of $\tau $_Dx_ and radii over time are estimated.

(C) Quantify the molecular stoichiometry within condensates. Raw photon data is binned into 1-ms bins to generate fluorescence trajectories. By defining the threshold as three standard deviations (SDs) above the mean, only bursts exceeding the threshold are selected to calculate intensity ratio (*I*_AF488_/*I*_Cy5_), which is converted into the molecular composition (*N*_KKETPV_/*N*_PDZ_) after correcting relative intensity and background.

(D) Quantify the binding affinity of the client within condensates. Amplitudes of autocorrelation and cross-correlation curves are calculated and their ratios are used to estimate the binding constant of the client within condensates.

## EXPERIMENTAL DESIGN

### Sample preparation

dcFCCS is a fluorescence based method, which requires signals from two fluorophores of different wavelengths. It is necessary to choose a suitable dye with minimal crosstalk between them for dcFCCS measurements. We recommend AF488 and Cy5 (or fluorophores of similar spectra). The crosstalk of AF488 signal into the Cy5 detection channel is ~1%. However, the crosstalk of Cy3 signal into the Cy5 detection channel is 5%–6%.

Proteins and nucleic acids can be labeled via different chemical procedures. Both ySmF derived proteins, KKETPV_14_ and PDZ_14_, contain an engineered cysteine residue to site-specifically react with maleimide derived fluorophores. The reaction between NHS ester derived fluorophores and NH_2_ group is another common way to label proteins. Fluorescent proteins fused proteins are suitable for dcFCCS measurements. Labeled short DNA and RNA can be customized synthesized.

Please pay attention to the influence of labeling on the aggregation and activity of the protein. Sometimes, excess dye or high labeling temperature induces aggregation of the protein. Therefore, size-exclusion chromatography is recommended to remove aggregated proteins before dcFCCS assays. A monomeric variant of GFP (A206K) is recommended to avoid oligomerization induced by GFP (Alberti* et al.* 2018). In addition, specific tags, such as Q3 tag (GQQQLG) (Lin and Ting 2006) and A1 tag (GDSLDMLEWSLM) (Zhou* et al.* 2007), are alternative labeling approaches to label proteins.

### Passivated slides preparation

PEG-passivated slides are prepared to reduce non-specific adsorption of biomolecules and condensates on slide surface, which decreases signals and increases background and noise. If needed, 0.1%–1% BSA or 0.02%–0.1% Tween-20 (Bi* et al.* 2016) can be included to further reduce non-specific adsorption. However, whether BSA and Tween-20 affect LLPS needs to be examined case-by-case. The fluorescent background contributed by the buffer is usually 10–500 Hz. If the background is higher than expected, buffer and coverslips need to be replaced by a fresh clean batch.

### dcFCCS calibration

Before performing the experiment, it is necessary to calibrate the instrument using a standard fluorophore and a dual-labeled dsDNA, to estimate the excitation volumes of two lasers and their overlapped volume. The excitation volume of 488 nm laser is determined by a standard fluorophore, such as AF488, whose diffusion coefficient is known. The value of the excitation volume is an important indicator, which is usually around 1 fL. Its sudden increase indicates misalignment of the microscope, which needs to be adjusted before further measurements. Correction factor *Cd*_488_ is used to calibrate diffusion times extracted from autocorrelation curves and cross-correlation curves. Correction factors *Cr*_488_ and *Cr*_640_ are the ratios of the overlapped excitation volume over the excitation volume of 488 nm laser and 640 nm laser, respectively. Usually, values of *Cr*_488_ and *Cr*_640_ are ~0.5, otherwise, the instrument needs to be maintained to increase overlapping between 488 nm and 640 nm lasers.

### Data acquisition

Before the experiment, confirm all optical components including dichroic mirrors and the bandpass filters are suitable for selected fluorophores. Our experiment is based on a home-built confocal microscope. Commercial microscope, such as FV 1200 laser scanning confocal microscope equipped with avalanche photodiode detectors (APDs), is also able to perform dcFCCS measurements.

Quantitative interpretation of FCS results strictly depends on the geometrical shape of the confocal excitation volume from which fluctuating fluorescence signals are detected. Due to reflection index mismatch between immersion oil and an aqueous sample, the water microscope objective is recommended for quantifying concentrations of labeled species in solution. However, in this procedure, molecular stoichiometry and relative concentration ratio of labeled species are quantified without quantifying their concentrations. As a result, the confocal microscope equipped with an oil immersion objective is also suitable for such applications.

After mixing, the growth of condensates formed from AF488-KKETPV_14_ and Cy5-PDZ_14_ can take hours. Therefore, dcFCCS curves and raw photon data can be collected for at least 1 h for this model system. According to our own experience, the formation of nanoscale condensates is usually faster than what we expect based on the results of conventional optical microscopy. Therefore, we recommend to start data collection right after mixing all necessary components to initiate LLPS.

### dcFCCS data analysis

This step is data mining. The first step is to extract amplitudes and diffusion times from correlation curves. Different models are applied under different conditions. Autocorrelation curves of AF488-KKETPV_14_ and Cy5-PDZ_14_, respectively, are fit by Eq. 1, which includes three-dimensional (3D) diffusion and the triplet state of a single component.

1 $ G\left(\tau \right)=A\dfrac{1}{\left(1+\dfrac{\tau }{{\tau }_{\mathrm{D}}}\right)}\dfrac{1}{\sqrt{\left(1+\dfrac{\tau }{{{a}^{2}\tau }_{\mathrm{D}}}\right)}}\left(1+\dfrac{T}{1-T}\cdot {e}^{-\tfrac{\tau }{{\tau }_{\mathrm{t}\mathrm{r}\mathrm{i}}}}\right) , $

in which *A* is the amplitude of the autocorrelation curve (*A*_488_ and *A*_640_), $ {\tau }_{\mathrm{D}} $ is the diffusion time of the labeled molecules, *T* is the triplet-state fraction and $ {\tau }_{\mathrm{t}\mathrm{r}\mathrm{i}} $ is the relaxation time of the triplet state. For the confocal volume from which fluorescence signals were collected for FCS analysis, *a* is the ratio of the vertical radius of the confocal volume over its horizontal radius. *A* is the *G*(0) value of the auto-correlation curves, and *G*(0) = 1/*N* with *N* being the average number of molecules within the confocal volume.

Cross-correlation curve (also known as dcFCCS curve) between AF488-KKETPV_14_ and Cy5-PDZ_14_, is fit by 3D diffusion model of a single component, shown in Eq. 2.

2 $ {G}_{x}\left(\tau \right)={A}_{x}\dfrac{1}{\left(1+\dfrac{\tau }{{\tau }_{\mathrm{D}\mathrm{x}}}\right)}\dfrac{1}{\sqrt{\left(1+\dfrac{\tau }{{{a}^{2}\tau }_{\mathrm{D}\mathrm{x}}}\right)}} , $

in which *A*_x_ is the amplitude of the cross-correlation curve, that is the *G*_x_(0) value of the cross-correlation curve, and $ {\tau }_{\mathrm{D}\mathrm{x}} $ is the diffusion time of the dual-labeled molecules.

The fraction of AF488-KKETPV_14_ forming dual-labeled molecules is calculated by

3 $ \frac{{N}_{x}}{{N}_{488}}=\frac{{A}_{x}}{{A}_{640}\;\times\; {Cr}_{640}} , $

and the fraction of Cy5-PDZ_14_ forming dual-labeled molecules is calculated by

4 $ \frac{{N}_{x}}{{N}_{640}}=\frac{{A}_{x}}{{A}_{488}\times {Cr}_{488}} , $

correction factor *Cr*_488_ and *Cr*_640_ are determined in Step 8(F).

When quantifying stoichiometry, it is important to set a threshold so that bursts and background are well separated. In KKETPV_14_ and PDZ_14_ phase separation system, the threshold is defined as three SDs above the mean. Thresholds from two SDs above the mean to four SDs above the mean are also tested, displaying minor effects on the intensity ratio (*I*_AF488_/*I*_Cy5_). Users should adjust the threshold value in their own measurements.

## PROCEDURE

### Sample preparation [TIMING 3–24 h]

1　Prepare labeling system. Maleimide derived fluorophores and cysteine-containing ySmF variants are usually mixed at 5:1–10:1 molar ratio in 50 mmol/L Tris-HCl pH 7.5, 150 mmol/L NaCl and 1 mmol/L TCEP. AF488-maleimide dye is used to label ySmF-KKETPV_14_ and Cy5-maleimide dye is used to label ySmF-PDZ_14_.

[**? TROUBLESHOOTING**]

2　Incubate the reaction mixtures at room temperature for 2 h.

[**? TROUBLESHOOTING**]

3　Separate the excess free fluorophores from labeled protein by NAP-5 column.

[**? TROUBLESHOOTING**]

4　According to the extinction coefficient of protein and chemical dye, labeling efficiency of KKETPV_14_ and PDZ_14_ can be calculated by absorbance values in 280 nm, 495 nm and 652 nm, which is determined by UV-VIS spectrometers. Because both the protein and commonly used dye contribute to light absorbance at 280 nm. It is necessary to use UV-VIS spectrometers to measure the extinction coefficient of chemical dye at 280 nm, which is used to calculate the corrected *A*_280_ value contributed only by the protein. Some manufacturer of chemical dye provides its extinction coefficient at 280 nm. Labeling efficiency of KKETPV_14_ is calculated via $ \dfrac{{A}_{495}/{\varepsilon }_{{\rm{AF}}488}}{{\mathrm{C}\mathrm{o}\mathrm{r}\mathrm{r}\mathrm{e}\mathrm{c}\mathrm{t}\mathrm{e}\mathrm{d}\;A}_{280}/{\varepsilon }_{{\rm{KKETPV}}}} $, and labeling efficiency of PDZ_14_ is calculated via $ \dfrac{{A}_{652}/{\varepsilon }_{{\rm{Cy}}5}}{{\mathrm{C}\mathrm{o}\mathrm{r}\mathrm{r}\mathrm{e}\mathrm{c}\mathrm{t}\mathrm{e}\mathrm{d}\;A}_{280}/{\varepsilon }_{{\rm{PDZ}}}} $, in which *ε*_AF488_, *ε*_Cy5,_ *ε*_KKETPV_ and *ε*_PDZ_ are the molar extinction coefficients of AF488 in 495 nm, Cy5 in 652 nm, KKETPV in 280 nm and PDZ in 280 nm, respectively.

[**CRITICAL STEP**] It is important to prepare the well-behaved labeling sample without aggregation or oligomerization while maintaining its activity. Labeling conditions, such as temperature, time, molar ratio of dye *vs.* protein and purification procedure after labeling, can all be adjusted to optimize the labeling process. Different labeling procedures, including NHS derived dye, A1 and Q3 tags, and fusing monomeric fluorescent protein with the protein of interest, can be used.

[**? TROUBLESHOOTING**]

5　After labeling, aliquot the AF488-KKETPV_14_ and Cy5-PDZ_14_ (~10 μL per tube) and store at −80 °C after flash-frozen.

[**CRITICAL STEP**] Repeated freezing and thawing cycles may influence protein quality or phase separation ability. Therefore, prepare small aliquots to avoid repeated freezing and thawing.

[**? TROUBLESHOOTING**]

### Passivated slide preparation [TIMING ~2 d]

6　Prepare PEG-passivated slides to avoid adsorption of protein during dcFCCS measurements (Ha* et al.* 2002; Peng* et al.* 2017).

[**? TROUBLESHOOTING**]

(A) At 40 °C, slides and coverslips are first sonicated by ethanol for 10 min, then 0.2 mol/L KOH for 20 min and ethanol for another 10 min.

(B) Incubate dried cleaned slides and coverslips with amino-silane solution containing 1% 3-aminopropyltriethoxysilane, 4% acetic acid and 95% methanol at room temperature overnight.

[**CRITICAL STEP**] When cleaned slides and coverslips are incubated with amino-silane solution, it is necessary to avoid water, which would disrupt the reaction and influence the quality of slides.

(C) After washing away amino-silane solution, add 60–80 μL PEGylation solution (15 mg mPEG2000-SVA in 60 μL 0.1 mol/L sodium bicarbonate) on each slide with a pipette, then cover with a coverslip and incubate at room temperature for 2.5–3 h. Humidify the incubation boxes to prevent the dry of PEGylation solution.

(D) Wash the slides and coverslips with ultra-pure water and dry them with clean N_2_. Put a pair of coverslips and slide in a 50 mL centrifuge tube, vacuum seal in a plastic bag and store at −20 °C.

### dcFCCS calibration [TIMING 1–2 h]

7　Calibrate the excitation volume of dcFCCS with a standard dye sample.

(A) Turn on the microscope and run the operation software. Turn on the solid-state laser (488 nm) 30 min before measurements. Make sure a suitable dichroic mirror and emission bandpass filter are used for AF488.

(B) Add immersion oil to the oil immersion objective. Add clean water if a water immersion objective is used. Place the coverslips on the microscope stage and move the stage to the suitable position. Turn coarse and fine adjustment knobs to focus on the upper surface of coverslips. At that position, the laser point is the smallest. To focus on the solution, raise the focal point 10 μm above the upper surface.

(C) Before data acquisition, set the detecting parameter, single cross mode and 5 min detecting time.

(D) 10 nmol/L AF488 dye is used as a standard sample and applied to passivated coverslips.

(E) FCS curves and raw photon data are recorded for 5 min under 488 nm laser excitation (~5 μW after the objective).

(F) Autocorrelation curves of AF488 are fitted by a one-component 3D diffusion model with a triplet state (Eq. 1), from which amplitude and diffusion time of AF488 dye are quantified. Excitation volume is then calculated by Eq. 5 (Bacia* et al.* 2014).

5 $ \qquad\quad {V}_{eff}=8\times a\times {(\pi \times D\times {\tau }_{D})}^{3/2}. $

8　Calibrate the correction factors of dcFCCS by dual-labeled DNA.

(A) 100% AF488 and Cy5 labeled DNA can be customized synthesized by companies, such as Sangon and IDT. Order 45-nt ssDNA labeled with AF488 fluorophore and 45-nt complementary ssDNA labeled with Cy5 fluorophore. In a 20 μL reaction system, mix two ssDNAs in 1:4 or 4:1 molar ratio to form dsDNA. The mixtures are gradually annealed from 75 °C to 20 °C at 1 °C/min and kept at 20 °C for an additional 10 min.

i. To ensure all AF488 ssDNA participating in dsDNA, 10 μmol/L AF488 ssDNA is mixed with 40 μmol/L Cy5 ssDNA, which is named as AF488-dsDNA.

ii. To ensure all Cy5 ssDNA participating in dsDNA, 40 μmol/L AF488 ssDNA is mixed with 10 μmol/L Cy5 ssDNA, which is named as Cy5-dsDNA.

(B) As Step 7(A), turn on solid-state lasers (488 nm and 640 nm). Set up the light path suitable for dual-labeled DNA detection. Fluorescence is split by T635lpxr dichroic mirror and further filtered by ET525/50m bandpass filter for AF488 channel and by ET700/75m for Cy5 channel before APDs.

[**CRITICAL STEP**] It is important to use suitable dichroic mirror and bandpass filters for optimized detection.

(C) As Step 7(B), place coverslips in the right position, raise the focal point in the solution and set parameters for data acquisition.

(D) Dilute AF488-dsDNA and Cy5-dsDNA to 10 nmol/L (based on concentrations of dsDNA) and add them to coverslips separately.

(E) Under 488 nm and 640 nm excitation (~5 μW after the objective), dcFCCS curves and raw photon data are recorded for 5 min.

(F) Autocorrelation curves are fitted with Eq. 1 and cross-correlation curves are fitted with Eq. 2 to determine their amplitudes and diffusion times.* Cr*_488_ is calculated via *A*_x_/*A*_488_, which are extracted from data of Cy5-dsDNA. *Cr*_640_ is calculated via *A*_x_/*A*_640_, which are extracted from data of AF488-dsDNA. Correction factor *Cd*_488_ is calculated via $ {\tau }_{488}/{\tau }_{x} $, which are extracted from data of AF488-dsDNA (Werner* et al.* 2018).

### Data acquisition [TIMING 2–3 d]

9　After sample labeling and instrument calibration, it’s time to acquire the data.

(A) Repeat Steps 7(A)**–**7(C). Turn on the solid-state lasers (488 nm and 640 nm). Set up the right light path, including T635lpxr dichroic mirror, ET525/50m and ET700/75m bandpass filters and APDs. Place coverslips in the right position and focus on the solution by turning the coarse and fine adjustment knobs.

(B) Observe 10 nmol/L AF488-KKETPV_14_ and Cy5-PDZ_14_ separately. Record FCS curves and raw photon data for 10 nmol/L AF488-KKETPV_14_ under 488 nm laser illumination (~5 μW after the objective) and for 10 nmol/L Cy5-PDZ_14_ under 640 nm laser illumination (~5 μW after the objective). 5-min data collection is usually enough and can be extended if needed. This step is to determine the molecular brightness of AF488-KKETPV_14_ and Cy5-PDZ_14_ (details in the section “Data analysis” below), respectively.

(C) In 20 μL reaction buffer (50 mmol/L Tris-HCl pH 7.5, 150 mmol/L NaCl, 1 mmol/L TCEP), mix 1 nmol/L AF488-KKETPV_14_ with 1 nmol/L Cy5-PDZ_14_ together. Add the 20 μL mixture to passivated coverslips. Record dcFCCS curves and raw photon data for 9 min under 488 nm and 640 nm laser excitation.

(D) After incubating for additional 41 min, record dcFCCS curves and raw photon data for another 9 min under 488 nm and 640 nm laser excitation. Users should adjust recording and incubation times based on the behaviors of their LLPS systems.

[**? TROUBLESHOOTING**]

10 To generate a phase diagram, in 20 μL reaction buffer (50 mmol/L Tris-HCl pH 7.5, 150 mmol/L NaCl, 1 mmol/L TCEP), mix AF488-KKETPV_14_ and Cy5-PDZ_14_ at different concentration combinations. Repeat Steps 9(C)–9(D). In our previous manuscript (Peng* et al.* 2020), a 9 × 9 concentration matrix of AF488-KKETPV_14_ and Cy5-PDZ_14_ is examined, whose concentrations range from 1 nmol/L to 500 nmol/L.

11 To detect the binding affinity of the client with condensates, phase separated PRM_14_ and Cy5-(SH3-KKETPV)_14_ condensates are used to recruit AF488-PDZ monomer. In 20 μL reaction buffer (50 mmol/L Tris-HCl pH 7.5, 150 mmol/L NaCl, 1 mmol/L TCEP), mix 400 nmol/L PRM_14_ and 200 nmol/L Cy5-(SH3-KKETPV)_14_ together to induce phase separation.

12 200 nmol/L client molecules AF488-PDZ is added into PRM_14_-Cy5-(SH3-KKETPV)_14_ mixture. Add the whole reaction mixture to the coverslips and record dcFCCS curves and raw photon data for 5 min under 488 nm and 640 nm laser excitation.

### Data analysis [TIMING 1–2 d]

13 dcFCCS curves and raw photon data are used to extract various physical and chemical properties of condensates, including the size and growth rate of condensates, stoichiometry within condensates as well as the binding affinity of client molecules with condensates.

(A) Quantify the size of condensates. In Steps 9–10, dcFCCS curves have been acquired. According to Eq. 2, dcFCCS curves are fitted to extract diffusion times ($ {\tau }_{\mathrm{D}\mathrm{x}} $), which can be used to quantify hydrodynamic radii of heterocomplexes and condensates formed from AF488-KKETPV_14_ and Cy5-PDZ_14_ ($ {R}_{\mathrm{c}\mathrm{o}\mathrm{n}\mathrm{d}} $) via Eq. 6.

6 $ \qquad\quad{R}_{\mathrm{c}\mathrm{o}\mathrm{n}\mathrm{d}}={R}_{\mathrm{d}\mathrm{y}\mathrm{e}}\times \frac{{\tau }_{\mathrm{D}\mathrm{x}}\times {Cd}_{488}}{{\tau }_{\mathrm{d}\mathrm{y}\mathrm{e}}} , $

here, $ {\tau }_{\mathrm{D}\mathrm{x}} $ of heterocomplexes and condensates and $ {\tau }_{\mathrm{d}\mathrm{y}\mathrm{e}} $ of AF488 have been determined in Steps 9(C)–9(D) and Step 7(F), respectively. *R*_dye_ of AF488 is 0.58 nm according to published results (Heyman and Burt 2008).

[**? TROUBLESHOOTING**]

(B) Quantify the growth rate of condensates. The 9-min-length raw data is divided into nine 1-min-length data. Like Step 13(A), for each 1-min-length data, calculate and fit autocorrelation curves with Eq. 1 and fit cross-correlation curves with Eq. 2. Values of *A*_488_, *A*_640_ and *A*_x_, and $ {\tau }_{\mathrm{D}\mathrm{x}} $ of dcFCCS curves for each 1-min-length data are all quantified.

i. Fraction of AF488 labeled molecules participating into condensates formation is calculated via Eq. 3.

ii. Fraction of Cy5 labeled molecules participating into condensates formation is calculated via Eq. 4.

iii. $ {\tau }_{\mathrm{D}\mathrm{x}} $ indicates the size of condensates. It is also used to quantify dynamics of condensates growth over time from the changes of hydrodynamic radii calculated from 1-min-length data via Eq. 6.

(C) Quantify the molecular stoichiometry within condensates. Raw photon data is binned into 1-ms bins to generate fluorescence trajectories.

i. To convert the intensity ratio into molecular ratio, the brightness of individual molecules needs to be determined. In Step 9(C), autocorrelation curves of AF488-KKETPV_14_ and Cy5-PDZ_14_ have been acquired, respectively, from which *A*_488_ and *A*_640_ are determined. Molecular brightness of AF488-KKETPV_14_ (*Q*_AF488_) and Cy5-PDZ_14_ (*Q*_Cy5_) can be estimated.

7 $\qquad\qquad
Q_{{\rm{AF}}488}={A}_{488}\times {I}_{488}$

8 $\qquad\qquad
Q_{{\rm{Cy}}5} ={A}_{{\rm{Cy}}5}\times {I}_{{\rm{Cy}}5} $

in which *I*_488_ and *I*_Cy5_ are the fluorescence intensities collected in the AF488 and Cy5 detection channels, respectively, which are usually defined as the number of photons collected per ms. In our system, *Q*_AF488_ of AF488-KKETPV_14_ is 16.8 ± 0.4 counts per ms, and *Q*_Cy5_ of Cy5-PDZ_14_ is 9.2 ± 0.6 counts per ms.

ii. Defining the threshold as three SDs above the mean, only bursts exceeding the threshold in both AF488 and Cy5 detection channels are selected to calculate the intensity ratio (*I*_AF488_/*I*_Cy5_) after subtracting of the background. The molecular composition (*N*_KKETPV_/*N*_PDZ_) of each burst can be estimated via Eq. 9.

9 $ \qquad\qquad\frac{{N}_{{\rm{KKETPV}}}}{{N}_{{\rm{PDZ}}}}=\frac{{I}_{{\rm{AF}}488}/{Q}_{{\rm{AF}}488}}{{I}_{{\rm{Cy}}5}/{Q}_{{\rm{Cy}}5}}, $

plot the distribution of log(*N*_KKETPV_/*N*_PDZ_) of all bursts and fit it with Gaussian function to determine the peak center, from which averaged molecular stoichiometry within condensates is estimated.

[**CRITICAL STEP**] Defining the suitable threshold is an important step to select bursts to calculate the molecular composition within condensates. In the previous study, we also tested 2 SDs above the mean and 4 SDs above the mean, which have minor effects on the intensity ratio (*I*_AF488_/*I*_Cy5_). In different phase separation systems, it is necessary to adjust the threshold value to optimize the burst selection.

(D) Quantify the binding affinity of client molecules with condensates. In Steps 11–12, client AF488-PDZ is added to bind with PRM_14_-Cy5-(SH3-KKETPV)_14_ condensates, and raw data is acquired. Like Step 13(A), fit autocorrelation curves and cross-correlation curves to calculate *A*_x_ and *A*_640_. Corrected *A*_x_/*A*_640_ indicates the proportion of client molecules (AF488-PDZ) binding with condensates. *c*_AF488-PDZ_ and *c*_KKETPV_ are initial concentration of AF488-PDZ and initial effective concentration of KKETPV, respectively, which are usually similar to each other. [AF488-PDZ-KKETPV] is the concentration of AF488-PDZ bound with condensates calculated via Corrected *A*_x_/*A*_640_ $ \times $ *c*_AF488-PDZ_. Then binding constant can be calculated via Eq. 10.

10 $ \qquad\quad\begin{split} {K}_{d}=\;&\{({c}_{{\rm{AF}}488{\text{-}}{\rm{PDZ}}}-\left[{\rm{AF}}488{\text{-}}{\rm{PDZ}}{\text{-}}{\rm{KKETPV}}\right])\times \\&({c}_{{\rm{KKETPV}}}-\left[{\rm{AF}}488{\text{-}}{\rm{PDZ}}{\text{-}}{\rm{KKETPV}}\right])\}/\\&{[{\rm{AF}}488{\text{-}}{\rm{PDZ}}{\text{-}}{\rm{KKETPV}}]}. \end{split}\end{equation*}

[**? TROUBLESHOOTING**]

Troubleshooting advice can be found in Table 1.

**Table 1:** Troubleshooting table

Step	Problem	Possible reason	Solution
1–4	Low labeling efficiency	Short labeling time or low labeling temperature or low dye concentration	Extending labeling time, raising labeling temperature, improving salt concentration of labeling buffer, or improving dye concentration are helpful for optimizing labeling efficiency.
1–4	Protein aggregation	High temperature or excessive free dye induces protein into unstable state and into aggregation at last.	Shorten labeling time or lower dye concentration is helpful for homogenous state of protein. Size-exclusion chromatography is also an alternative way to remove aggregated protein from labeling product.
1–4	Activity of protein is diminished dramatically after labeling	Active center of protein may be disrupted by the labeling.	Try different labeling procedures, including NHS derived dye, A1 and Q3 tags, and fusing monomeric fluorescent protein with the protein of interest.
5	Strong adsorption of protein onto storing tubes.	Proteins may non-specifically stick to tube walls.	Try protein low-binding tubes.
6	Strong adsorption of protein onto coverslips	Proteins and condensates may non-specifically bind to passivated coverslips.	Prepare a new batch of coverslips. Try to add 0.1%–1% mg/mL BSA or 0.02%–0.1% Tween-20 in buffer to reduce adsorption.
9(D)	Buffer evaporation after long observation time	Long observation time results in evaporation of water.	Add oil above the buffer to prevent evaporation. In our experiments, we used immersion oil (Cat. No. 16245, Cargille Laboratories).
13(A)	One-component 3D diffusion model is not suitable for fitting auto or cross-correlation curves.	Size distributions of condensates are broad and deviate from the one-component mode.	Two-component diffusion model and anomalous diffusion model can be used.

[**TIMING**]

Steps 1–5, sample preparation: 3–24 h

Step 6, passivated slide preparation: ~2 d

Steps 7–8, dcFCCS calibration: 1–2 h

Steps 9–12, data acquisition: 2–3 d

Step 13, data analysis: 1–2 d

## ANTICIPATED RESULTS

**Figure 1:**
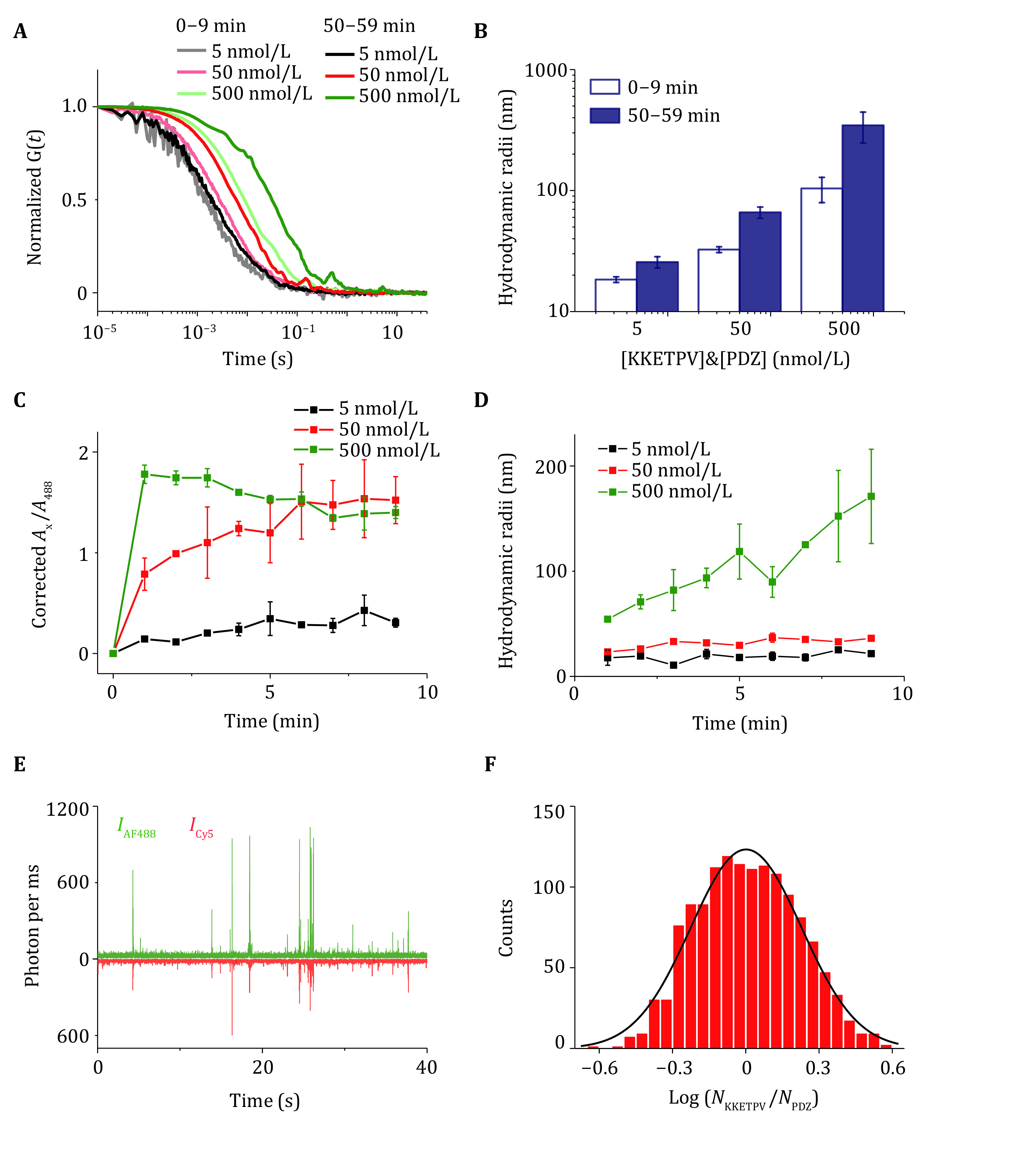
Quantification of KKETPV_14_-PDZ_14_ heterocomplexes via dcFCCS. **A** Normalized cross-correlation curves between AF488-KKETPV_14_ and Cy5-PDZ_14_ taken right after mixing and after incubation for 50 min. Concentrations of ySmF monomer are listed. **B** Hydrodynamic radii of KKETPV_14_-PDZ_14_ heterocomplexes right after mixing (open) and after incubation for 50 min (solid). **C** Increase of corrected *A*_x_/*A*_488_ in the first 9 min after mixing. **D** Increase of the size of KKETPV14-PDZ14 heterocomplexes in the first 9 min. **E** 1-ms-binned fluorescence trajectory of a mixture of 50 nmol/L AF448-KKETPV_14_ and Cy5-PDZ_14_. **F** Distribution of molecular stoichiometry extracted from the fluorescence trajectory

We generated two ySmF variants (KKETPV_14_ and PDZ_14_) (Zhou* et al.* 2020) to induce LLPS. KKETPV_14_ and PDZ_14_ are labeled by maleimide-derived AF488 and Cy5 fluorophores, as shown in Steps 1–5. After mixing AF488-KKETPV_14_ and Cy5-PDZ_14_, dcFCCS experiments were performed to detect dual-labeled condensates whose diffusion times correlate with molecular weight, as shown in Steps 8–9 (Fig. 1A). When high concentrations of KKETPV_14_ and PDZ_14_ were used, diffusion time of dcFCCS curves increased (Fig. 1A). Longer incubation time leads to larger condensates and longer diffusion time (Fig. 1A). After data analysis, as shown in Step 13(A), diffusion time of dcFCCS was converted into hydrodynamic radii (Fig. 1B).

To examine the dynamics of phase separation formation, growth rates of condensates were calculated, as shown in Step 13(B). 9-min-length raw data was divided into nine 1-min-length data. Fractions of components and condensates radii were plotted over time to quantify the growth rate of condensates (Fig. 1C, 1D). To quantify the stoichiometry, as shown in Step 13(C), bursts above the background were identified and selected from 1-ms-binned fluorescent trajectory (Fig. 1E). After correcting relative intensity and background, distributions of KKETPV_14_/PDZ_14_ molecular composition within each burst were fitted by Gaussian distributions to determine the molecular stoichiometry (Fig. 1F).

**Figure 2:**
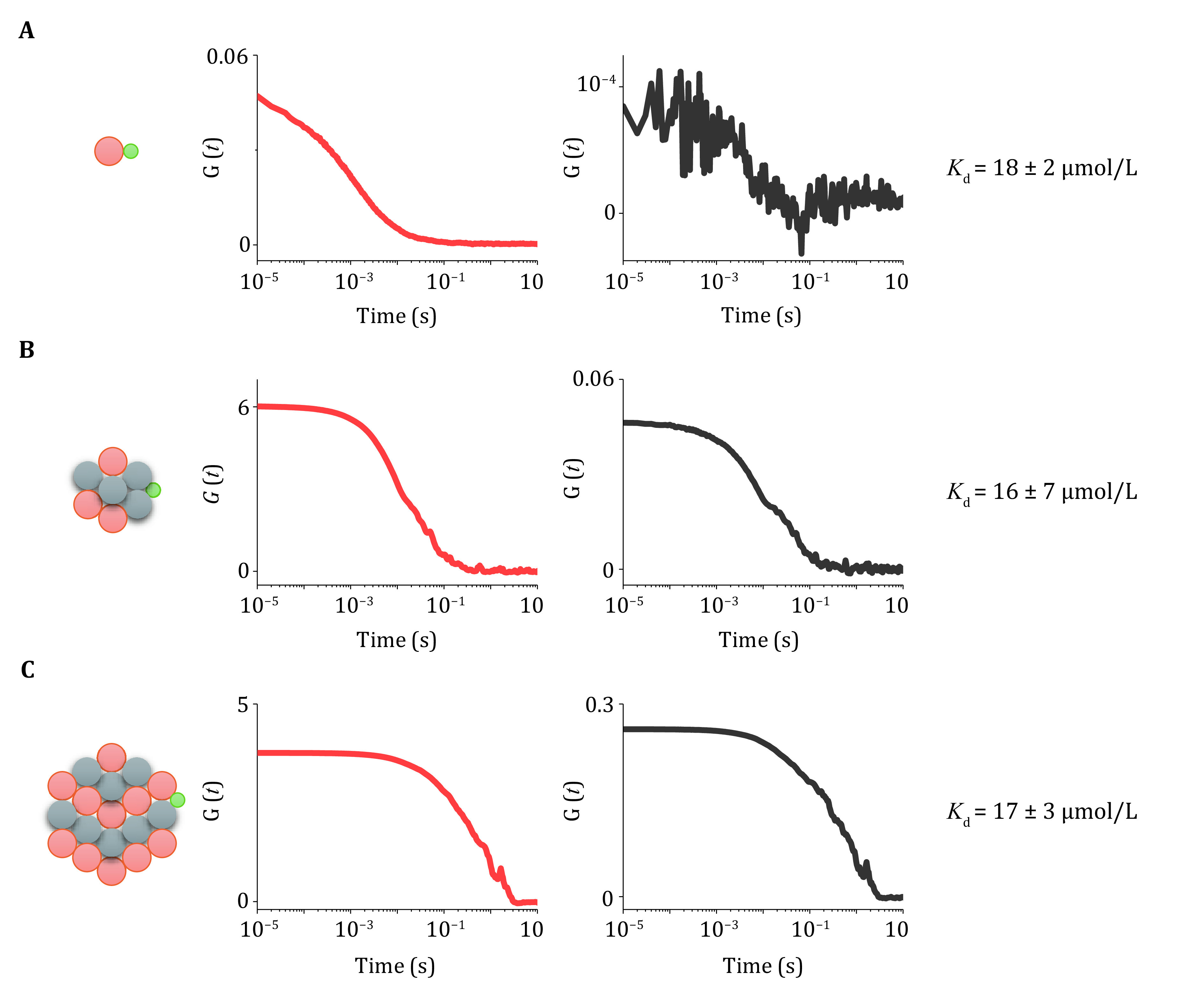
Quantification of binding affinity between client and condensates. **A**–**C** Autocorrelation curves of Cy5 channel (red) and cross-correlation curves (black) between AF488 and Cy5 under different conditions. 200 nmol/L AF488-PDZ monomer interacted with 200 nmol/L Cy5-(SH3-KKETPV)_14_ (**A**), 200 nmol/L Cy5-(SH3-KKETPV)_14_ and 400 nmol/L PRM_14_ (**B**), and 2 μmol/L Cy5-(SH3-KKETPV)_14_ and 4 μmol/L PRM_14_ (**C**), respectively

To quantify the binding affinity of client protein within condensates, another phase separation system was generated. It is formed from unlabeled PRM_14_ and Cy5-(SH3-KKETPV)_14_, which is able to recruit AF488-PDZ. Then dcFCCS experiment was performed, like Step 12. After data acquisition, auto-correlation and cross-correlation curves were obtained between Cy5-PRM_14_-(SH3-KKETPV)_14_ condensates and AF488-PDZ to calculate the binding affinity (Fig. 2A, 2C). As shown in Step 13(D), based on concentrations of components and corresponding participating proportions, binding affinities (*K*_d_) between PDZ and KKETPV in the absence of phase separation and in the presence of condensates of different sizes were quantified.

## MATERIALS

### Reagents

• Alexa Fluor^TM^ 488 C_5_ maleimide (Invitrogen, cat. no. A10254)

• Sulfo Cyanine5 maleimide (Lumiprobe, cat. no. 13380)

• UltraPure^TM^ 1 mol/L Tris-HCl pH 7.5 (Invitrogen, cat. no. 15567027)

• Nuclease-free water (Invitrogen, cat. no. AM9932)

• 5 mol/L NaCl (Invitrogen, cat. no. AM9760G)

• Tris (2-carboxyethyl) phosphine hydrochloride (TCEP) (Goldbio, cat. no. 51805-45-9)

• Ethanol (Fisher Chemical, cat. no. A995-4)

[**CAUTION!**] Ethanol is unstably explosive. Avoid flames.

• KOH (Sigma, cat. no. 1310-58-3)

[**CAUTION!**] KOH is corrosive and irritant. Handle with protective gloves.

• 3-aminopropyltriethoxysilane (Energy Chemical, cat. no. 919-30-2)

• Acetic acid (Fisher Chemical, A35-500)

[**CAUTION!**] Acetic acid is volatile. Handle in fume hood.

• Methanol (Fisher Chemical, cat. no. A452-4)

[**CAUTION!**] Methanol is acutely toxic and unstably explosive. Avoid flames and handle in a fume hood. Dispose in accordance with local regulations.

• mPEG-Succinimidyl Valerate (Laysan Bio Inc., cat. no. MPEG-SVA-2000)

• Sodium bicarbonate (Sigma-Aldrich, cat. no. 127-09-3)

### Reagent setup

#### Reaction Buffer (50 mmol/L Tris-HCl pH 7.5, 150 mmol/L NaCl, 1 mmol/L TCEP)

Add 5 mL 1 mol/L Tris-HCl (pH 7.5) and 3 mL 5 mol/L NaCl into 92 mL nuclease-free water. Dissolve 0.0286 g TCEP in 100 mL buffer. Aliquot as 1 mL buffer per tube and store at −20°C.

#### KOH (0.2 mol/L)

Dissolve 11.22 g KOH in 1000 mL deionized water. This solution can be stored at room temperature for two months.

[**CAUTION!**] KOH is corrosive and irritant. Handle with protective gloves.

#### Amino-silane reagent

Add 1 mL 3-aminopropyltriethoxysilane and 5 mL acetic acid into 94 mL methanol. Prepare the solution freshly every time.

[**CAUTION!**] Acetic acid is volatile. Handle in a fume hood.

#### PEGylation solution

Dissolve 60 mg mPEG2000 in 240 μL 0.1 mol/L sodium bicarbonate. Prepare fresh solution every time and add them onto coverslips within minutes.

### Equipments

• NanoDrop^TM^ (Thermo Scientific)

• Mastercycler nexusGX (Eppendorf)

• Ultrasonic cleaner (Xinzhi, SB-120DT&5L)

• NAP^TM^-5 column (GE healthcare, 17-0853-01)

### Abbreviations

AF488 Alexa Fluor 488

APD　 　 Avalanche photodiode detector

CLEM　 Correlated light and electron microscopy

Cy5　 Cyanine5

dcFCCS　 Dual-color fluorescence cross-correlation 　　　　 spectroscopy

dsDNA　 Double-stranded DNA

FCS　 Fluorescence correlation spectroscopy

FUS Fused in sarcoma

LLPS　 Liquid-liquid phase separation

PEG　 Polyethylene glycol

ssDNA　 Single-stranded DNA

STORM　 Stochastic optical reconstruction microscopy

TCEP　 Tris (2-carboxyethyl) phosphine 　　　　 hydrochloride

## Conflict of interest

Yirong Yao, Wenjuan Wang and Chunlai Chen declare that they have no conflict of interest.
